# A New Source of Data for Public Health Surveillance: Facebook Likes

**DOI:** 10.2196/jmir.3970

**Published:** 2015-04-20

**Authors:** Steven Gittelman, Victor Lange, Carol A Gotway Crawford, Catherine A Okoro, Eugene Lieb, Satvinder S Dhingra, Elaine Trimarchi

**Affiliations:** ^1^Mktg, IncEast Islip, NYUnited States; ^2^USDA National Agricultural Statistics ServiceResearch and Development DivisionWashington, DCUnited States; ^3^National Center for Chronic Disease and Health PromotionDivision of Population HealthCenter for Disease Control and PreventionAtlanta, GAUnited States; ^4^Custom Decision SupportLos Angeles, CAUnited States; ^5^Northrop GrummanAtlanta, GAUnited States

**Keywords:** big data, social networks, surveillance, chronic illness

## Abstract

**Background:**

Investigation into personal health has become focused on conditions at an increasingly local level, while response rates have declined and complicated the process of collecting data at an individual level. Simultaneously, social media data have exploded in availability and have been shown to correlate with the prevalence of certain health conditions.

**Objective:**

Facebook likes may be a source of digital data that can complement traditional public health surveillance systems and provide data at a local level. We explored the use of Facebook likes as potential predictors of health outcomes and their behavioral determinants.

**Methods:**

We performed principal components and regression analyses to examine the predictive qualities of Facebook likes with regard to mortality, diseases, and lifestyle behaviors in 214 counties across the United States and 61 of 67 counties in Florida. These results were compared with those obtainable from a demographic model. Health data were obtained from both the 2010 and 2011 Behavioral Risk Factor Surveillance System (BRFSS) and mortality data were obtained from the National Vital Statistics System.

**Results:**

Facebook likes added significant value in predicting most examined health outcomes and behaviors even when controlling for age, race, and socioeconomic status, with model fit improvements (adjusted *R* ^2^) of an average of 58% across models for 13 different health-related metrics over basic sociodemographic models. Small area data were not available in sufficient abundance to test the accuracy of the model in estimating health conditions in less populated markets, but initial analysis using data from Florida showed a strong model fit for obesity data (adjusted *R* ^2^=.77).

**Conclusions:**

Facebook likes provide estimates for examined health outcomes and health behaviors that are comparable to those obtained from the BRFSS. Online sources may provide more reliable, timely, and cost-effective county-level data than that obtainable from traditional public health surveillance systems as well as serve as an adjunct to those systems.

## Introduction

The development of the Internet and the explosion of social media have provided many new opportunities for health surveillance. The use of the Internet for personal health and participatory health research has exploded, largely due to the availability of online resources and health care information technology applications [1-8]. These online developments, plus a demand for more timely, widely available, and cost-effective data, have led to new ways epidemiological data are collected, such as digital disease surveillance and Internet surveys [8-25]. Over the past 2 decades, Internet technology has been used to identify disease outbreaks, track the spread of infectious disease, monitor self-care practices among those with chronic conditions, and to assess, respond, and evaluate natural and artificial disasters at a population level [6,8,11,12,14,15,17,22,26-28]. Use of these modern communication tools for public health surveillance has proven to be less costly and more timely than traditional population surveillance modes (eg, mail surveys, telephone surveys, and face-to-face household surveys).

The Internet has spawned several sources of big data, such as Facebook [29], Twitter [30], Instagram [31], Tumblr [32], Google [33], and Amazon [34]. These online communication channels and market places provide a wealth of passively collected data that may be mined for purposes of public health, such as sociodemographic characteristics, lifestyle behaviors, and social and cultural constructs. Moreover, researchers have demonstrated that these digital data sources can be used to predict otherwise unavailable information, such as sociodemographic characteristics among anonymous Internet users [35-38]. For example, Goel et al [36] found no difference by demographic characteristics in the usage of social media and email. However, the frequency with which individuals accessed the Web for news, health care, and research was a predictor of gender, race/ethnicity, and educational attainment, potentially providing useful targeting information based on ethnicity and income [36]. Integrating these big data sources into the practice of public health surveillance is vital to move the field of epidemiology into the 21st century as called for in the 2012 US “Big Data Research and Development Initiative” [19,39].

Understanding how big data can be used to predict lifestyle behavior and health-related data is a step toward the use of these electronic data sources for epidemiologic needs [36,40]. Facebook has been used by individuals and public health researchers for novel surveillance applications [13,37,38,41-44]. For example, Chunara et al [13] used Facebook to examine the association between activity- and sedentary-related likes and population obesity prevalence. These researchers found that populations with higher proportions of activity-related Facebook likes had a lower prevalence of being overweight and/or obese. Facebook likes are a means by which Facebook users can identify their own preferred Internet sites and interests. Although Facebook likes are not explicitly health-related, researchers have shown that when taken together, the “network” of an individual’s likes are predictive of sociodemographic characteristics, health behaviors, obesity, and health outcomes [13,37,42,44]. Timian et al [44] examined whether Facebook likes for a hospital could be used to evaluate 2 quality measures (ie, 30-day mortality rates and patient recommendations) both quickly and inexpensively. Facebook likes have also been shown to be predictors of a variety of user attributes, such as intelligence, happiness, race, religious and political views, sexual orientation, and a spectrum of personality traits [37]. Researchers have proposed that Facebook likes be used as a new behavioral measure in a fashion similar to traditional questionnaires [37].

In this study, we focused on harnessing the predictive power of Facebook likes for enhancing population health surveillance. Toward this end, we viewed Facebook likes as a class of big data that may help us understand population health at a local level. Given that risk factors and associated health outcomes are often clustered in populations geographically [10,45,46], the ability to identify, monitor, and intervene at a community level exists. Although past research has used specific categories of likes to target theoretically related conditions (eg, [13]), it is possible that the entirety of the Facebook dataset can be used to form a complete profile of individuals that can be broadly applied to predictive models in a number of areas. If the Facebook characteristics of a region can predict physical activity, smoking, and self-management of chronic disease, then a strong argument can be made in favor of using these data to target, monitor, and intervene on adverse lifestyle behaviors.

In this paper, we examine how big data might be used to complement traditional surveillance systems. We explored the use of Facebook likes as potential predictors of health outcomes and the behavioral determinants of poor health outcomes at the county level. Specifically, we hypothesized that (1) Facebook likes provide a means of predicting county-level mortality, (2) Facebook likes can be used as an indicator of chronic disease outcomes (obesity, diabetes, and heart disease) that contribute to increased mortality, and (3) Facebook likes can be used as an indicator of adverse lifestyle behaviors that impact disease. If these hypotheses hold, then Facebook likes could ultimately be used to enhance population health surveillance.

## Methods

### Data Sources

Data for the analysis were collected from 4 sources. Objective reports on key health indicators (ie, life expectancy, mortality, and low birth weight) were collected from the National Vital Statistics System (NVSS) for 2011, which provides population data on deaths and births in the United States. According to its website, “these data are provided through contracts between [National Center for Health Statistics] NCHS and vital registration systems operated in the various jurisdictions legally responsible for the registration of vital events—births, deaths, marriages, divorces, and fetal deaths” [47].

Self-reported health outcome and risk behavior data were obtained from the Behavioral Risk Factor Surveillance System (BRFSS) [48]. The BRFSS is an ongoing random digit-dialed telephone survey operated by state health agencies with assistance from the Centers for Disease Control and Prevention (CDC). The surveillance system collects data on many of the behaviors and conditions that place adults aged ≥18 years at risk for chronic disease, disability, and death. The large sample size of the 2011 BRFSS (N=506,467) facilitated the calculation of reliable estimates for 214 counties with 500 or more respondents. In addition, the 2010 BRFSS facilitated the calculation of reliable estimates for 91% of counties in Florida—a year in which 61 of its 67 counties had 500 or more respondents. County-level risk factor data were obtained from the 2011 Selected Metropolitan/Micropolitan Area Risk Trends (SMART) BRFSS [49].

Facebook likes data were collected using the Facebook advertising application program interface (API) [50] in February 2013, which aggregates the number of users by zip code who expressed a positive inclination (“like”) toward certain categories of items by zip code. These zip code data were aggregated to the county level to allow for direct comparisons to the health data, with zip codes crossing borders assigned to the county they predominantly rest in. The data reflect the cumulative total of Facebook users’ likes at the time they were drawn. Out of 8 supercategories of available Facebook likes (ie, events, family status, job status, activities, mobile device owners, interests, Hispanic, and retail and shopping), 3 were deemed as potentially correlated with health and were selected for the model. The selected likes were activities, interests, and retail and shopping. These supercategories were selected because they contained items with an explicit theoretical relationship to health. For example, “interests” contains the “health and well-being” category, to which the relationship of health is self-explanatory. The “activities” category was chosen because it included “outdoor fitness and activities,” which seemed directly applicable to measures of physical activity, whereas “retail and shopping” was chosen due to its apparent linkage to socioeconomic status, a powerful driver of health outcomes (Multimedia Appendix 1) [51,52].

All constituent elements of these supercategories were used, regardless of a clear relationship to health, because the exact contents and means of construction of these data are not reported by Facebook. Other supercategories lacked these explicit links, although we acknowledge the possibility that potentially powerful indirect relationships may exist. Due to rounding performed automatically by the API that routinely led to overestimates, counties with fewer than 1000 profiles overall were excluded from the analysis. Facebook likes for each category were scored as a percentage of completed profiles in an area. Finally, to reduce multicollinearity caused by variation in levels of Facebook usage by county, values were divided by the average percentage of likes across all categories. The resulting variables can be characterized as a measure of popularity for each category relative to that of other categories. Although the individual variables resulting from this transformation were sometimes entirely uncorrelated with the originals, estimates using the raw and transformed variables correlated at *R*=.9. Thus, we concluded that the results of the proceeding analyses were not an artifact of this transformation.

Population data, such as average income, median age, and sex ratio, were collected using the 2010 US Census [53] and broken into county aggregates. Supporting county-level statistics unrelated to health were collected using “USA Counties Information” provided by the Census Bureau [54]. Overall, 214 counties in the continental United States contained sufficient data on all variables in the analysis.

### Variables of Interest

Several sociodemographic, health outcome, and risk factor variables were selected for analysis. These included income, age, education, employment, nonwhite population, obesity, diabetes, physical activity, and smoking, as well as other measures such as general health status. A comprehensive listing, as well as the data source and assessment of each variable of interest are available in Multimedia Appendix 2.

### Data Analysis

We began by using principal components analysis on the 37 Facebook likes categories within the 3 selected supercategories as a data reduction technique. We then used these factors in an ordinary least squares (OLS) regression to determine whether Facebook likes could predict a number of health outcomes, conditions, and related behaviors. Finally, by limiting our analysis to Florida, where available data were more comprehensive, we formed a predictive model via bootstrap regression [55] that demonstrated the predictive accuracy of Facebook in a visual format.

## Results

The first stage in the analysis was to establish that health outcomes could indeed be determined by Facebook likes. Through principal components analysis, the 37 categories were reduced to 9 factors (varimax rotation) purely as a means of simplifying modeling efforts by reducing these categories into the latent sociobehavioral dimensions we believed they represented. This number was arrived on by applying the Cattell scree test (shown in Multimedia Appendix 3) [56], which evaluates the “elbow” in the distribution of eigenvalues; that is, the point at which additional factors do not seem to provide a substantial gain in variance explained. Each factor is numbered in accordance with the amount of variance it explains (Multimedia Appendix 4). Any attempt to interpret the actual nature of these factors is subject to errors in the interpretation of the Facebook advertising data; as such, we avoided the urge to do so. However, the factor loadings of each of the categories can be seen in Multimedia Appendix 5.

To test our hypothesis that Facebook likes can be used to predict mortality on their own, we used OLS regression. We used the 9 Facebook factors to predict life expectancy, with no other controls included in the initial model. The results, as shown in the “Facebook only” column of Table 1, were quite strong (model adjusted *R* ^2=^.69). Despite this relationship, Facebook only has value insofar as it provides predictive value beyond that of reliable data that is already available through the census or other means. Regression results for an OLS model predicting life expectancy with demographic information (average age and nonwhite population) and socioeconomic status (SES; as represented by average household income, unemployment rate, and percentage with bachelor’s degree) are shown in the “SES only” column of Table 1. There is a very strong relationship to be found there as well, although it is less strong than for Facebook factors alone. Finally, the 2 groups of variables are combined in the last column of Table 1, indicating that although a great deal of the variance in life expectancy is shared by both the Facebook and SES variables, the addition of Facebook improves the model fit above and beyond readily available socioeconomic measures. The resulting adjusted *R* ^*2*^=.81 also indicates that a considerable amount of the variation in county-level life expectancy can be explained by SES and Facebook likes.

**Table 1:** Ordinary least squares regression coefficients (β) for life expectancy (all independent variables are standardized).

		Facebook only		SES only		Facebook and SES	
		β	*P*	β	*P*	β	*P*
**Facebook factor**							
	1	–0.14	<.001	—	—	0.20	<.001
	2	0.79	<.001	—	—	0.43	<.001
	3	–0.96	<.001	—	—	–0.30	<.001
	4	0.60	<.001	—	—	0.42	<.001
	5	0.69	<.001	—	—	0.41	<.001
	6	0.21	<.001	—	—	–0.04	.05
	7	–0.08	<.001	—	—	–0.04	.04
	8	–0.61	<.001	—	—	–0.49	<.001
	9	0.12	<.001	—	—	0.10	.70
Age		—	—	0.16	<.001	0.01	.87
Income		—	—	0.62	<.001	0.59	<.001
Education		—	—	0.88	<.001	0.61	<.001
Unemployment		—	—	–0.05	0.07	0.01	.70
Nonwhite population		—	—	–0.85	<.001	–0.47	<.001
Constant		77.08	<.001	77.06	<.001	77.06	<.001
Adjusted *R* ^*2*^		.69		.64		.81	
RMSE		1.28		1.29		1.01	

Table 2 summarizes regressions using the same set of predictors run across an array of health-related dependent variables and indicates the percent improvement in variance explained by the inclusion of Facebook likes when added to SES compared to the SES alone. There are 2 conclusions we can draw from this model. First, Facebook likes and SES in tandem prove to be effective predictors of all tested disease outcomes. Second, there is a persistent benefit of Facebook likes beyond that contributed by SES, although its magnitude varies widely.

Our third hypothesis posited that Facebook likes, as a measure of behavior, should be able to determine the behaviors that drive health outcomes. The results in Table 2 clearly show that Facebook likes had a sizeable impact in the predictive models of all tested health-related behaviors and in some cases, such as health insurance and exercise, the total model fit was quite strong.

**Table 2:** Facebook likes impact on model fit for 214 counties.

Dependent variable	Source^a^	Facebook, *R* ^*2*^	SES, *R* ^*2*^	SES + Facebook, *R* ^*2*^	Improvement with Facebook, %
Life expectancy	NVSS	.69	.64	.81	27%
Mortality	NVSS	.57	.49	.60	22%
Low birthweight	NVSS	.53	.17	.57	235%
Obesity	BRFSS	.46	.56	.60	7%
Diabetes	BRFSS	.36	.39	.55	41%
Heart attack	BRFSS	.32	.46	.46	0%
Stroke	BRFSS	.27	.30	.41	46%
Exercise	BRFSS	.57	.51	.76	49%
Insured	BRFSS	.48	.37	.65	76%
Self-Reported health	BRFSS	.51	.20	.55	175%
Smoker	BRFSS	.40	.42	.54	29%
Last checkup	BRFSS	.69	.30	.72	140%
Declined treatment	BRFSS	.39	.35	.49	40%

^a^ BRFSS: Behavioral Risk Factor Surveillance System; NVSS: National Vital Statistics System.

### Predicting Health Conditions

The natural extension of these findings would be to map out predicted prevalence of health conditions in data-deficient counties. Although 214 counties were sampled sufficiently for the BRFSS to provide county-specific estimates, the remaining 2895 counties were not. An additional source of data, such as Facebook, would be a cost-effective way to augment existing state-level data sources that are used to produce county-level estimates, such as the BRFSS.

However, attempting to apply predictions nationally from the 2011 SMART data creates a problem. Although predictions correlate well with actual levels in non-SMART data, mean levels are consistently upwardly biased. We hypothesized that the selection method that leads counties to be weighted according to the SMART program creates a nonrepresentative sample with better levels of general health than we see in the United States in general, particularly in areas that are more rural. As an alternative without such problematic selection issues, we limited our predictive model to 2010 Florida data. Florida collects more than 500 interviews in 61 of its 67 counties every 3 years, leading to a dataset that has neither sample size shortages nor selection biases relative to the state at large.

Using data exclusively from one state creates its own problems for a predictive model. Although the integrity of the data is very good, there is no easy way to correct for the various cultural differences between Florida and other states. Attempting to apply Florida-based models to the full set of SMART counties results in only fair level of correlation (*R*=.63). Although it indicates that relationships exist, this is not a sufficient level of accuracy on which to base policy decisions. Instead, we have limited our analysis to Florida to demonstrate the level of accuracy we feel can be achieved at a national level once a somewhat more representative selection of county-level data are made available.

The results of a predictive model are shown in Table 3. These are the results of a bootstrap regression procedure in which 50 observations were drawn over 100 replications. Standard errors are high due to the limited sample size, but 2 of our Facebook likes categories retain their significance in the model. Although we would expect demographics and socioeconomic data to be very effective at predicting “healthy” versus “unhealthy” communities, we believe that the additional information provided by Facebook likes should help to clarify the finer distinctions between communities with similar general levels of health.

**Table 3:** Ordinary least squares regression (β) results for prediction of obesity.

Header		Facebook only		SES only		Facebook and SES	
		β	*P*	β	*P*	β	*P*
**Facebook factor**							
	1	0.04	.05	—	—	–0.03	<.001
	2	–0.02	.06	—	—	–0.01	.14
	3	0.03	<.001	—	—	–0.01	.07
	4	–0.02	.06	—	—	–0.01	.74
	5	–0.02	.04	—	—	0.03	.01
	6	–0.02	.07	—	—	–0.02	.13
	7	–0.05	.30	—	—	0.02	.04
	8	0.01	.34	—	—	0.01	.90
	9	0.02	.36	—	—	–0.01	.17
Age		—	—	–0.01	.01	–0.01	.01
Income		—	—	–0.01	.37	–0.01	.59
Education		—	—	–0.03	<.001	0.01	.35
Unemployment		—	—	–0.01	.04	0.01	.58
Nonwhite population				0.02	.04		
Constant		0.29	<.001	0.30	<.001	0.30	<.001
Adjusted *R* ^*2*^		.77		.72		.8	
RMSE		0.03		0.03		0.03	

**Figure 1:**
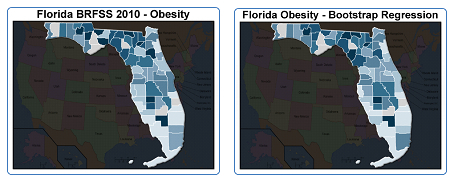
Actual statistics compared with predicted values for obesity, 2010 BRFSS. Darker colors represent higher prevalence. Light gray indicates missing data.

Figure 1 shows a graphical comparison of predicted values from the bootstrap regression procedure versus source data for obesity in Florida, where nearly all counties were sufficiently sampled for reliable estimates. These maps are dynamically shaded from light to dark in accordance with the level of obesity, with data separated into septiles of prevalence. As should be apparent visually, the fit is generally good—90% of errors in the model fall inside of ±2.1% (0.4 standard deviations) from Florida’s estimated values from the 2010 BRFSS.

## Discussion

When we first undertook this research plan, it was our expectation that the larger part of the measurement error that would affect our results would come through the imprecise categorization and geographic aggregation of the Facebook data. However, although there are some exceptions, the consistency and strength of fit we have found seem manifest. Our models do extremely well in predicting levels of health variables across counties where data are plentiful, and often diverge from BRFSS estimates where they are not. This suggests the possibility that data imputed from Facebook and vital statistics may provide a more accurate picture in small counties than the current methodology that aggregates data across several years.

Thus, we argue that Facebook can serve an intermediary role in augmenting sparse data at a community level. We have shown that it can do so already, but additional health survey data, especially in less extensively measured regions (eg, rural), could only help. Although complete measurement is unfeasible and would render the Facebook modeling moot, ensuring that communities of all types are represented in sufficient number when estimating the model is a necessary step in avoiding the risk of systematic error in its predictions.

The ultimate goal of our analysis of Facebook likes is to establish the potential contribution of big data to research that directly affects government spending and public policy, and—most importantly—contributes to improved population health. At a fraction of the cost of traditional research, data that might seem on its face to have little to do with health can predict epidemic-level health problems such as diabetes and obesity. With the need to augment traditional public health surveillance systems with readily available, cost-effective, and geographically relevant health data, the use of “big epidemiologic data” comes at just the right time.

The nature of the Facebook data source prevents it from being a useful tool in several situations. In the case of very small counties (approximately 9% of the total) and in smaller geographic areas, rounding error becomes so great that estimates cannot be reliably used, even though they may be provided by Facebook. Additionally, Facebook profiles are untested as a tool for tracking the prevalence of infectious diseases. They may be better suited to predicting endemic and ongoing conditions that are unlikely to fluctuate over the course of short time periods.

Further, some might find it counterintuitive that Facebook data are being used to “predict” health data that not only predates it, but to which it is not causally related through any theoretical mechanism. Likes data for a given geographic area should be viewed as a product of sociobehavioral conditions within that region in the same manner that health outcomes are. As such, the likes data can be viewed as an instrument for those conditions, which are causally linked. Although the temporal concerns are not ideal, they are not especially problematic because those health metrics used in this research are not especially prone to fluctuation over short time periods.

Finally, without a clear insight into the manner in which the categories of Facebook likes are constructed and by which individuals are tagged as being interested in a given category, it is difficult to achieve more nuanced insights into the relationships between social network behavior and health outcomes. Unless Facebook becomes more transparent regarding the ways in which these data are compiled, they will remain a “black box” and we must take on faith that the interests and activities being measured are indeed those it claims to measure.

The relationships examined here demonstrate that social media may hold promise to be used as an indicator of local conditions, even those that have little relationship to the activity that takes place on Facebook. As we predicted, significant relationships that extend beyond the predictive power of local demographics exist between an area’s aggregate Facebook behavior and the incidence of diseases and of adverse lifestyle behaviors that very well may lead to those diseases.

We have also indicated the severe shortage of health data that are available in most American counties. Although Facebook data may not reach into every corner of the United States, it seems an effective enough tool to augment the existing county-level data in the majority of counties. With demand for local health data growing, such tools seem far more cost-effective than an increase in survey surveillance regardless of the mode through which it might be conducted.

Whether this data ultimately comes from Facebook or not is of little importance. The online landscape may change and it may provide a different source of data that proves more viable in the future. So long as the source reflects people’s activities in daily life, the same relationships may hold. Even if Facebook does prove to endure as a social institution, however, there is still room for a great deal of improvement on the models presented here. With cooperation from the social media outlets themselves, we may be able to obtain better estimates in categories that align better with our needs. In the end, our data may not suffer because of the rising costs of research. Instead, exploring newly opened avenues of data collection online could lead to more reliable, timely, and cost-effective county-level data than that obtainable from traditional public health surveillance systems as well as serve as an adjunct to those systems.

## Multimedia Appendix 1

Facebook category structure.

## Multimedia Appendix 2

Demographic variable descriptions.

## Multimedia Appendix 3

Scree plot for principal components analysis.

## Multimedia Appendix 4

Rotated (orthogonal varimax) factors.

## Multimedia Appendix 5

Factor loadings.

## Abbreviations

API: application program interface
BRFSS: Behavioral Risk Factor Surveillance System
NVSS: National Vital Statistics System
OLS: ordinary least squares
SES: socioeconomic status
SMART: Selected Metropolitan/Micropolitan Area Risk Trends
