# Supplementary material for: A New Source of Data for Public Health Surveillance: Facebook Likes
Source: J Med Internet Res. 2015 Apr 20;17(4):e98. doi: 10.2196/jmir.3970 (PMC4419195; doi:10.2196/jmir.3970)
Supplement: Supplementary file 1 [file jmir_v17i4e98_app1.pdf]

Appendix 1: Facebook Category Structure

	Supercategories		
	**Interests**	**Activities**	**Retail & Shopping**
Categories	Auto Intenders	Charity	Beauty Products
	Automotive	Cooking	Fashion
	Beer, Wine & Spirits	Dancing	Luxury Goods
	Electronics	Do-it-Yourselfing	
	Environment	Teaching	
	Planning	Television	
	Health & Wellness	Food & Dining	
	Home and Garden	Fast Food	
	Literature	Frequent Diner	
	News	Social Gaming	
	Pets	Gaming Consoles	
	Cats	Gardening	
	Dogs	Outdoor Fitness	
	Politics	Photography	
	Conservative Politics	Photo Uploading	
	Liberal Politics	Travel	
	Non-partisan Politics		
	Pop Culture		
