# Supplementary material for: A New Source of Data for Public Health Surveillance: Facebook Likes
Source: J Med Internet Res. 2015 Apr 20;17(4):e98. doi: 10.2196/jmir.3970 (PMC4419195; doi:10.2196/jmir.3970)
Supplement: Supplementary file 2 [file jmir_v17i4e98_app2.pdf]

Appendix 2: Demographic Variable Descriptions

Control Variables	Source	Question Wording or Description
Average Household Income	2010 Census	Mark the "Yes" box for each income source received during 2009 to a maximum of $999,999.
Median Age	2010 Census	What is this person's age and what is this person's date of birth?
Percent with bachelors degree	2010 Census	What is the highest degree or level of school this person has COMPLETED?
Percent non-white population	2010 Census	What is this person's race or origin?
% Unemployed	Bureau of Labor Statistics (2010)	% in Labor Force without a job
Obesity	BRFSS 2011 (SMART)	Body mass index > 30 based on self-reported height and weight
Diabetes	BRFSS 2011 (SMART)	Have you ever been told by a doctor that you have diabetes? (Gestational diabetes excluded)
Physically Inactive	BRFSS 2011 (SMART)	No to: During the past month, other than your regular job, did you participate in any physical activities or exercises such as running, calisthenics, golf, gardening, or walking for exercise?
Uninsured	BRFSS 2011 (SMART)	No to: Do you currently have health insurance?
Fair/Poor General Health	BRFSS 2011 (SMART)	In general, would you say your health is Excellent, Very Good, Good, Fair or Poor?
Smokes Every Day	BRFSS 2011 (SMART)	(To those who have smoked 100 cigarettes) Do you now smoke cigarettes every day, some days, or not at all?
Last Checkup	BRFSS 2011 (SMART)	About how long has it been since you last visited a doctor for a routine checkup?
Cost Barrier to Needed Healthcare	BRFSS 2011 (SMART)	Was there a time in the past 12 months when you needed to see a doctor but could not because of cost?
Heart Attack	BRFSS 2011 (SMART)	Has a doctor, nurse, or other health professional ever told you that you had a heart attack, also called a myocardial infarction?
Stroke	BRFSS 2011 (SMART)	Has a doctor, nurse, or other health professional ever told you that you had a stroke?
