# Supplementary figures and images for: A New Source of Data for Public Health Surveillance: Facebook Likes
Source: J Med Internet Res. 2015 Apr 20;17(4):e98. doi: 10.2196/jmir.3970 (PMC4419195; doi:10.2196/jmir.3970)

**Appendix 3: Scree Plot for Principal Components Analysis**

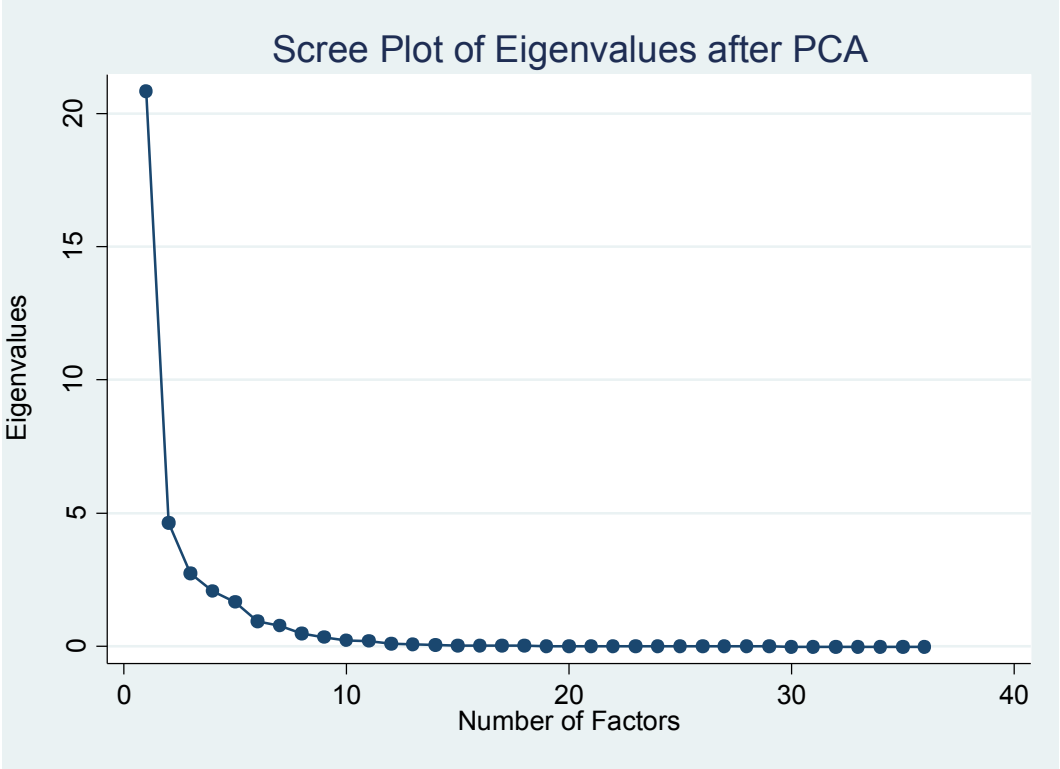

Supplement: Supplementary file 3 [file jmir_v17i4e98_app3.pdf]
