# Supplementary material for: A New Source of Data for Public Health Surveillance: Facebook Likes
Source: J Med Internet Res. 2015 Apr 20;17(4):e98. doi: 10.2196/jmir.3970 (PMC4419195; doi:10.2196/jmir.3970)
Supplement: Supplementary file 4 [file jmir_v17i4e98_app4.pdf]

Appendix 4: Rotated (Orthogonal Varimax) Factors

	Eigenvalue	Difference	Proportion	Cumulative %
Factor1	8.16	3.39	0.22	0.22
Factor2	4.77	0.80	0.13	0.35
Factor3	3.96	0.49	0.11	0.46
Factor4	3.48	0.39	0.09	0.55
Factor5	3.09	0.13	0.08	0.63
Factor6	2.93	0.43	0.08	0.71
Factor7	2.53	0.99	0.07	0.78
Factor8	1.54	0.51	0.04	0.82
Factor9	1.03	.	0.03	0.85
