# Supplementary material for: A New Source of Data for Public Health Surveillance: Facebook Likes
Source: J Med Internet Res. 2015 Apr 20;17(4):e98. doi: 10.2196/jmir.3970 (PMC4419195; doi:10.2196/jmir.3970)
Supplement: Supplementary file 5 [file jmir_v17i4e98_app5.pdf]

Appendix 5: Factor Loadings

Variable	Factor1	Factor2	Factor3	Factor4	Factor5
Auto Intenders	0.96	-0.02	0.05	-0.03	0.01
Automotive	0.37	0.18	0.50	0.45	0.28
Beauty Products	0.54	-0.18	0.51	-0.21	-0.26
Beer, Wine & Spirits	0.21	0.73	0.10	0.04	0.08
Charity	-0.11	-0.81	0.24	-0.04	-0.15
Electronics	-0.40	-0.07	0.76	-0.18	-0.19
Cooking	-0.02	0.34	-0.20	0.12	0.24
Dancing	0.21	-0.05	0.02	-0.09	-0.01
Do-it-Yourselfing	0.23	0.26	-0.06	0.48	0.34
Teaching	0.85	-0.09	-0.05	-0.13	-0.04
Television	-0.67	0.33	0.12	0.06	0.24
Environment	0.21	0.33	-0.33	0.63	0.41
Planning	0.96	-0.04	0.04	-0.04	0.01
Fashion	-0.64	-0.20	0.42	-0.22	-0.10
Fast Food	0.11	-0.14	0.09	-0.08	-0.93
Food & Dining	0.04	0.01	0.01	-0.01	0.093
Frequent Diner	-0.11	-0.16	0.02	-0.06	-0.93
Gaming Consoles	0.05	0.29	0.49	-0.04	0.04
Social Gaming	-0.03	0.08	0.82	0.23	0.05
Gardening	0.81	0.03	-0.01	0.33	0.19
Health & Wellness	0.01	0.44	-0.45	0.40	0.41
Home and Garden	0.17	0.19	0.09	0.27	0.28
Literature	-0.09	0.15	-0.16	0.47	0.39
Luxury Goods	0.34	0.10	-0.19	-0.78	-0.24
News	-0.33	0.32	-0.35	-0.16	0.16
Outdoor Fitness	-0.07	0.10	-0.17	0.19	0.13
Pets	0.11	0.53	0.09	0.31	0.20
Cats	0.92	0.17	0.10	-0.04	0.06
Dogs	0.77	0.41	0.08	0.15	0.14
Photo Uploading	0.80	0.05	0.02	-0.17	-0.20
Photography	0.78	0.15	-0.32	0.06	0.11
Politics	-0.20	-0.80	-0.09	-0.36	-0.04
Conservative Politics	-0.06	-0.79	-0.09	0.18	-0.19
Liberal Politics	-0.01	-0.24	-0.07	-0.89	0.12
Non-partisan Politics	-0.24	0.82	-0.04	0.27	0.12
Pop Culture	-0.39	0.24	0.52	0.08	-0.07
Travel	-0.22	0.05	-0.83	-0.07	0.05

Variable	Factor6	Factor7	Factor8	Factor9	Uniqueness
Auto Intenders	-0.07	-0.01	0.07	-0.07	0.06

Automotive	0.06	0.0489	-0.22	0.10	0.24
Beauty Products	0.06	0.1883	-0.24	0.14	0.20
Beer, Wine & Spirits	-0.16	0.3439	-0.05	-0.30	0.17
Charity	0.11	0.0665	-0.23	0.12	0.17
Electronics	-0.18	0.1409	0.07	0.16	0.11
Cooking	0.77	0.1273	0.15	-0.05	0.14
Dancing	-0.13	-0.9444	0.02	0.02	0.04
Do-it-Yourselfing	0.54	0.1425	0.23	-0.07	0.16
Teaching	-0.09	-0.4094	-0.06	-0.06	0.06
Television	0.26	0.2731	-0.26	0.01	0.15
Environment	0.15	0.0214	0.14	0.06	0.14
Planning	-0.07	-0.0121	0.05	-0.09	0.07
Fashion	-0.25	0.2634	0.13	-0.04	0.17
Fast Food	-0.13	0.0497	-0.10	0.05	0.06
Food & Dining	0.91	0.0815	0.06	-0.04	0.15
Frequent Diner	-0.14	0.08	-0.09	-0.05	0.05
Gaming Consoles	-0.20	0.06	0.23	0.64	0.17
Social Gaming	-0.15	0.11	0.14	0.15	0.18
Gardening	0.17	0.04	0.18	-0.16	0.11
Health & Wellness	0.29	0.09	0.10	0.06	0.16
Home and Garden	0.26	-0.04	0.79	0.13	0.07
Literature	0.47	0.08	-0.07	0.16	0.31
Luxury Goods	-0.04	0.12	-0.11	0.16	0.12
News	0.29	0.10	0.41	0.09	0.35
Outdoor Fitness	-0.05	-0.92	0.03	-0.05	0.05
Petse	0.38	0.14	-0.15	0.25	0.31
Cats	0.11	0.02	-0.01	0.11	0.09
Dogs	0.02	0.06	-0.06	0.20	0.14
Photo Uploading	0.02	0.03	-0.07	0.09	0.27
Photography	0.22	0.07	0.10	0.14	0.17
Politics	-0.23	0.17	-0.07	-0.24	0.04
Conservative Politics	-0.32	0.14	-0.03	-0.24	0.12
Liberal Politics	-0.01	0.05	-0.05	-0.04	0.12
Non-partisan Politics	0.16	0.05	-0.02	0.05	0.15
Pop Culture	0.06	0.31	-0.44	0.17	0.20
Travel	-0.12	0.06	0.09	0.15	0.20
